# Acute Pancreatitis Presenting With ST‐Segment Elevation and Marked Troponin Elevation: A Diagnostic Dilemma in a Resource‐Limited Setting—A Case Report

**DOI:** 10.1002/ccr3.73716

**Published:** 2026-10-11

**Authors:** Selamawit Kassahun Aweke, Natnael Mathewos Assale, Anteneh Girma Mengistu, Nathanael Elias Temesgen, Eden Fanta Amajo, Saron Yohannes Tsegaye, Merahi Kefyalew Merahi

**Affiliations:** ^1^ Addis Ababa University College of Health Sciences, Tikur Anbessa Hospital Addis Ababa Ethiopia; ^2^ Department of Emergency Medicine Addis Ababa University College of Health Sciences, Tikur Anbessa Hospital Addis Ababa Ethiopia; ^3^ Department of Emergency and Critical Care Medicine Addis Ababa University College of Health Sciences, Tikur Anbessa Hospital Addis Ababa Ethiopia

**Keywords:** acute pancreatitis, case report, electrocardiography, myocardial infarction, ST‐segment elevation, troponin

## Abstract

Acute pancreatitis can present with ST‐segment elevation and marked troponin elevation, mimicking acute coronary syndrome. In resource‐limited settings where coronary angiography, cardiac magnetic resonance imaging, and serial high‐sensitivity troponin testing are unavailable, distinguishing acute myocardial injury associated with pancreatitis from Type 1 myocardial infarction can be challenging.

## Key Clinical Message

Concurrent acute pancreatitis, ST‐segment elevation, and marked troponin elevation create profound diagnostic uncertainty. In resource‐limited settings where coronary angiography is inaccessible, Type 1 myocardial infarction cannot be definitively excluded; clinicians must integrate multimodal noninvasive findings and maintain vigilance for signs requiring urgent transfer to a catheterization‐capable facility.

**Abbreviations**
ACS: acute coronary syndrome
AI: artificial intelligence
AKI: acute kidney injury
ALP: alkaline phosphatase
AP: acute pancreatitis
ARDS: acute respiratory distress syndrome
AST: aspartate aminotransferase
BMI: body mass index
BNP: B‐type natriuretic peptide
BUN: blood urea nitrogen
CK‐MB: creatine kinase‐myocardial band
COPD: chronic obstructive pulmonary disease
CRP: C‐reactive protein
CT: computed tomography
ECG: electrocardiogram
ED: emergency department
ER: emergency room
ERCP: endoscopic retrograde cholangiopancreatography
GGT: gamma‐glutamyl transferase
ICU: intensive care unit
IV: intravenous
LAD: left anterior descending artery
LCx: left circumflex artery
LV: left ventricle/left ventricular
LVEF: left ventricular ejection fraction
MI: myocardial infarction
MINOCA: myocardial infarction with nonobstructive coronary arteries
NG: nasogastric
NPO: nil per os (nothing by mouth)
PCI: percutaneous coronary intervention
PND: paroxysmal nocturnal dyspnea
RCA: right coronary artery
SGLT2: sodium‐glucose cotransporter 2
SIRS: systemic inflammatory response syndrome
STEMI: ST‐elevation myocardial infarction
URL: upper reference limit

## 1 Introduction

Acute pancreatitis is characterized by inflammation of the pancreas, manifesting as abdominal pain and elevated pancreatic enzyme levels. The annual incidence ranges from 4.9 to 35 cases per 100,000 people [1]. The primary causes of acute pancreatitis are gallstones, accounting for 40%–70% of cases, and alcohol, responsible for 25%–35% of cases [2, 3]. Other contributing factors include hypertriglyceridemia, post‐ERCP complications, genetic predispositions, and certain medications.

Acute pancreatitis typically presents with severe, constant epigastric pain and elevated pancreatic enzyme levels. Possible complications include the formation of pancreatic pseudocysts, acute peripancreatic fluid collection, pancreatic necrosis, abdominal compartment syndrome, splanchnic venous thrombosis, and systemic complications such as deep vein thrombosis, acute respiratory distress syndrome (ARDS), acute kidney injury, shock, and exacerbation of pre‐existing cardiac conditions including heart failure and ischemic heart disease [4, 5, 6].

A mechanistic link between acute pancreatitis and myocardial injury is well described [7]. Pancreatitis can mimic acute coronary syndrome, with ECG abnormalities—including ST–T changes—present in approximately 25% of cases [8]. True ST‐segment elevation is exceedingly rare, creating a diagnostic and therapeutic dilemma: whether to treat ECG changes as a systemic manifestation of pancreatitis or to pursue aggressive ACS management. This report aims to enhance understanding of this rare association and its diagnostic challenges.

## 2 Case Presentation

A 62‐year‐old Ethiopian man presented to the Emergency Department (ED) with 5 days of semisolid diarrhea and mild chest pain. He initially visited a local health center, but the chest pain had progressively worsened, evolving into a squeezing, non‐radiating sensation that intensified with physical exertion. He subsequently developed shortness of breath, extreme fatigue, diaphoresis, and stabbing pain in the epigastric region and right upper quadrant, none of which radiated to other areas.

He had no history of orthopnea, paroxysmal nocturnal dyspnea (PND), lower extremity edema, or cough. He also reported no history of abdominal bloating, jaundice, bleeding tendencies, nausea, loss of appetite, vomiting, changes in bowel habits, or alterations in urinary output. There were no features of fever or other constitutional symptoms.

He had a 4‐year history of hypertension managed with 5 mg daily oral amlodipine, though adherence to medication and follow‐up had been poor, resulting in suboptimal blood pressure control. He had no prior history of diabetes mellitus, cardiac disease, renal disease, or chronic obstructive pulmonary disease (COPD). He had no known drug allergies; his body mass index was 31.2 kg/m^2^, consistent with obesity Class I (CDC Classification).

He regularly chewed khat and had consumed 2–5 bottles of alcohol daily (20%–30% alcohol content daily or per bottle) over the preceding 5 years. He was a farmer, married with children, and had no known family history of chronic diseases.

On physical examination, he was alert and oriented. Chest auscultation revealed fine bibasal crackles in the posterior lower lung fields. Abdominal examination demonstrated guarding in the upper abdomen and right upper quadrant, without clinically apparent ascites on initial assessment. All other system examinations—including head, eyes, ears, nose, throat, lymphatic, cardiovascular, gastrointestinal, musculoskeletal, integumentary, and central nervous systems—were unremarkable.

### 2.1 Laboratory Investigations Revealed

- White blood cell (WBC) count: 13.1 × 10^3^/μL (normal range: 4.0–10.0 × 10^3^/μL)—elevated (leukocytosis).
- Neutrophil count: 91.3% (normal range: 40%–75%)—elevated (neutrophilia).
- Platelet count: 102 × 10^3^/μL (normal range: 150–450 × 10^3^/μL)—decreased (thrombocytopenia).
- Hemoglobin level: 15 g/dL (normal range: 12–16 g/dL)—within normal limits.

### 2.2 Biochemical Investigations

- Aspartate aminotransferase (AST): 1.3‐fold above baseline—elevated.
- Alkaline phosphatase (ALP): 1.3‐fold above baseline—elevated.
- Lipase: 7.7‐fold above the upper reference limit—markedly elevated.
- Amylase: 7.5‐fold above the upper reference limit—markedly elevated.
- Creatinine: 2.06 mg/dL (normal range: 0.6–1.3 mg/dL)—elevated, consistent with acute kidney injury.
- Urea: 92 mg/dL (normal range: 7–20 mg/dL)—elevated.
- C‐reactive protein (CRP): 200 mg/L (normal range: < 5 mg/L)—markedly elevated.
- Sodium: 129.6 mmol/L (normal range: 135–145 mmol/L)—decreased (hyponatremia).
- Potassium: 3.64 mmol/L (normal range: 3.5–5.0 mmol/L)—within normal limits.
- Calcium: 0.84 mmol/L (normal range: 2.1–2.6 mmol/L for total calcium*)—decreased (hypocalcemia).
- Troponin I: 1.706 ng/mL (reference < 0.04 ng/mL)—markedly elevated, representing a 42.6‐fold increase above the 99th‐percentile upper reference limit.

### 2.3 Urinalysis

- Urine pH: 5.0 (normal range: 4.5–8.0)—acidic urine.
- Urine protein: +1 (normal: negative)—mild proteinuria.
- Urine sediment: Few granular casts—consistent with renal tubular injury.

### 2.4 Additional Investigations

BNP and CK‐MB were not obtained. Serial high‐sensitivity troponin measurements with a validated delta criterion were unavailable. Initial cardiac troponin I was measured via a conventional immunoassay (reference limit: < 0.04 ng/mL; limit of detection: 0.01 ng/mL), demonstrating marked elevation at 1.706 ng/mL on presentation. A repeat conventional troponin I obtained on Hospital Day 9 confirmed normalization to 0.04 ng/mL. While this pattern demonstrates a rise‐and‐fall trajectory consistent with acute myocardial injury under the Fourth Universal Definition of Myocardial Infarction, the absence of intermediate timed measurements limits kinetic analysis and does not by itself exclude a Type 1 ischemic event with spontaneous recanalization.

Echocardiography demonstrated mild left ventricular hypertrophy and mild systolic dysfunction (LVEF 45%–50%) without regional wall motion abnormalities in any coronary territory. No apical or mid‐ventricular ballooning pattern to suggest Takotsubo cardiomyopathy was identified, and there was no pericardial effusion. Abdominal ultrasound showed hepatomegaly with moderate fatty infiltration, moderate ascites, and a large complex peripancreatic fluid collection (initially described as pseudocyst but more accurately representing an acute peripancreatic fluid collection given the early disease course).

The ECG (Figure 1) obtained at presentation demonstrated sinus rhythm with a normal QRS axis and normal PR interval. ST‐segment elevation was present in leads V1, V2, and V3, estimated at 1–2 mm (exact millivolt measurements were not formally documented), without reciprocal ST depression in the inferior leads and without PR‐segment depression or diffuse saddle‐shaped morphology. These features raised concern for acute anteroseptal STEMI, though pericarditis was considered and deemed less likely based on ECG morphology. Serial ECG recordings were not consistently available during the admission, which limited assessment of ST‐segment evolution—an acknowledged diagnostic gap in this case.

**FIGURE 1:**
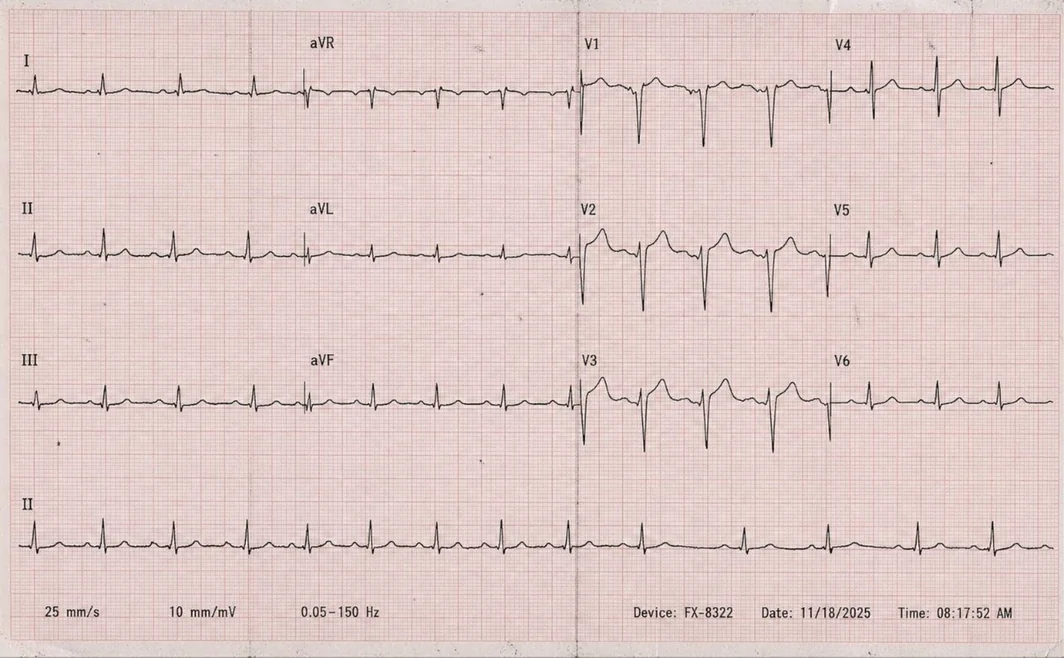
ST‐segment elevation is notably seen in leads V1, V2, and V3, suggestive of acute anteroseptal myocardial infarction (STEMI).

On arrival, the patient was initially managed for both suspected STEMI and acute pancreatitis (AP). He received supplemental oxygen (10–15 L/min), aspirin, clopidogrel, heparin, and morphine. Ceftriaxone 1 g IV BID was administered given concurrent leukocytosis, diarrhea, and concern for early sepsis, rather than as pancreatitis prophylaxis (not recommended in uncomplicated disease). He was subsequently admitted to the cardiac ICU, where intravenous (IV) fluid resuscitation was initiated and the patient was kept NPO. Atorvastatin was added to his regimen. IV calcium gluconate and potassium chloride were administered to correct electrolyte imbalances.

On the second day of hospitalization, the patient developed worsening abdominal distension accompanied by persistent bibasal crackles and peripheral fluid retention, raising clinical concern for secondary intravascular fluid overload following initial crystalloid resuscitation. Recognizing that diuretic use in early acute pancreatitis carries a risk of aggravating hypovolemia and worsening prerenal acute kidney injury, a cautious single low dose of intravenous furosemide (20 mg) was administered under strict fluid‐balance monitoring. Over the subsequent 48 h, the patient maintained adequate urine output (> 0.8 mL/kg/h), pulmonary crackles cleared, and serum creatinine steadily improved toward normal baseline, confirming renal recovery. Furosemide was discontinued once euvolemia was restored.

By the fourth day, the patient showed features consistent with partial large bowel obstruction. A nasogastric (NG) tube was inserted, and rectal stimulation was performed twice daily. Symptoms improved over the subsequent days.

Troponin subsequently normalized to 0.04 ng/mL, prompting transfer to medical ward and de‐escalation of MI‐directed therapy. Antidiabetic therapy was initiated with insulin; dapagliflozin was cautiously introduced after confirmed renal recovery. Pantoprazole was added. He was discharged after 19 days in stable condition, with all symptoms resolved. A chronological summary of key events, including symptoms, investigations, treatments, and outcomes, is shown in Table 1.

**TABLE 1:** Historical and current information of care organized as a timeline.

Hospital day	Clinical events	Management
Day 0	STEMI and acute pancreatitis	Initial ACS therapy, ceftriaxone, IV fluids, NPO, atorvastatin, electrolyte replacement; CICU
Day 2	Abdominal distension	IV furosemide
Day 4	Partial large bowel obstruction	NG tube, rectal stimulation
Days 5–7	Clinical improvement	Supportive care
Days 8–10	Troponin normalized	Medical ward transfer
Medical Ward Stay	Type 2 diabetes diagnosed	Continued cardiac therapy; insulin, dapagliflozin, pantoprazole
Day 19	Recovered	Discharged

## 3 Discussion

This case highlights the diagnostic difficulties when acute pancreatitis mimics myocardial infarction, particularly with concurrent gastrointestinal and cardiac presentations. Per the Revised Atlanta Classification, acute pancreatitis is defined by abdominal pain, elevated pancreatic enzymes, and compatible imaging findings [9]. Based on available data (elevated BUN, SIRS criteria met via leukocytosis and CRP), the patient likely met a BISAP score of ≥ 2, indicating moderately severe disease.

The initial ECG and elevated troponin were compatible with acute anterior STEMI, prompting early cardiology‐directed management. Markedly elevated lipase and amylase, hepatomegaly, peripancreatic fluid, and systemic inflammation established a concurrent diagnosis of acute pancreatitis [10]. Whether the troponin elevation reflected acute myocardial injury secondary to pancreatitis, Type 2 myocardial infarction from supply–demand mismatch, or Type 1 myocardial infarction from primary coronary plaque rupture or occlusion could not be definitively determined in this case. Troponin normalization without a documented pattern of ST‐segment evolution is compatible with, but does not establish, nonischemic myocardial injury, since spontaneous reperfusion of a transiently occluded coronary artery can produce an indistinguishable biochemical course. Because coronary angiography, serial ECGs, and validated serial high‐sensitivity troponin measurements were unavailable, Type 1 MI could not be excluded. This distinction is clinically important: Type 1 MI mandates urgent revascularisation, Type 2 MI directs treatment toward the precipitating cause, and nonischemic myocardial injury requires neither—so an imprecise label risks either undertreating a coronary event or overtreating a systemic one [11].

Prior reports describe ECG abnormalities in pancreatitis—including transient ST elevation—often without obstructive coronary disease [10]. Proposed mechanisms include systemic inflammation, electrolyte imbalance, microvascular dysfunction, and catecholamine surge. These same factors can precipitate Takotsubo cardiomyopathy, documented in association with acute pancreatitis [12, 13]; however, the absence of characteristic ballooning on echocardiography made this less likely, though it does not exclude Takotsubo syndrome, as ballooning can be transient or absent early in the disease course. Pericarditis was also considered, but the regionally confined ST elevation, absence of PR‐segment depression, and lack of pericardial effusion argued against it. The broader differential—encompassing MINOCA, myocarditis, Takotsubo, and pericarditis—could not be formally evaluated or excluded without coronary angiography or cardiac MRI, neither of which was available in this resource‐limited setting.

This dual presentation underscores the importance of a comprehensive diagnostic approach in which laboratory and imaging findings are interpreted in an integrated rather than isolated manner. Misinterpreting elevated troponin levels in the context of systemic inflammation could lead to unnecessary or potentially harmful treatments [14].

In a patient with persistent, convincing STEMI features, current guidelines support proceeding directly to urgent coronary angiography and primary PCI where these are available, rather than delaying for multimodal reassessment [14, 15]. In this case, ACS‐directed therapy was appropriately initiated in parallel with the pancreatitis work‐up, but coronary angiography could not be performed because the resource‐limited setting lacked catheterization capability. In this context, diagnostic decisions relied on serial clinical, ECG, biochemical, and echocardiographic reassessment rather than direct coronary imaging. Persistent or evolving ST‐segment elevation, recurrent ischemic symptoms, hemodynamic instability, or a rising rather than falling troponin should prompt urgent transfer to a PCI‐capable facility when feasible; resolving symptoms, a normalizing troponin, and a reassuring echocardiogram, as seen here, may support continued conservative management with close monitoring, while acknowledging that this remains an inference rather than a confirmed diagnosis.

Clinicians should consider acute pancreatitis as a potential diagnosis in patients presenting with atypical chest pain and elevated cardiac biomarkers, particularly when abdominal symptoms or risk factors such as alcohol use are present. The multisystem involvement in this case—evidenced by renal impairment, electrolyte disturbances, and hepatic abnormalities—underscores the need for prompt multidisciplinary collaboration [7].

Ultimately, this case demonstrates that in atypical STEMI presentations with concurrent systemic illness, continuous re‐evaluation and multidisciplinary collaboration are essential. Recognizing the overlap between acute pancreatitis and cardiac injury ensures accurate diagnosis, appropriate management, and improved patient outcomes.

## 4 Conclusion

Concurrent acute pancreatitis, ST‐segment elevation, and marked troponin elevation present a challenging diagnostic dilemma. In resource‐limited settings where coronary angiography and advanced cardiac imaging are unavailable, Type 1 myocardial infarction cannot be definitively excluded, and the cardiac findings should not be attributed solely to pancreatitis. Clinicians in resource‐limited environments must carefully weigh guideline‐directed reperfusion against conservative stabilization, remaining vigilant for high‐risk clinical triggers that warrant urgent transfer for invasive coronary intervention.

## Author Contributions

**Selamawit Kassahun Aweke:** writing – original draft, investigation, writing – review and editing. **Natnael Mathewos Assale:** writing – original draft, investigation, validation. **Anteneh Girma Mengistu:** conceptualization, writing – original draft, investigation, resources. **Nathanael Elias Temesgen:** conceptualization, writing – original draft, investigation, resources. **Eden Fanta Amajo:** conceptualization, writing – original draft, writing – review and editing. **Saron Yohannes Tsegaye:** writing – review and editing, investigation, writing – original draft. **Merahi Kefyalew Merahi:** writing – review and editing, conceptualization.

## Funding

The authors have nothing to report.

## Ethics Statement

Ethical approval is not required for a single anonymous case report at our institution.

## Consent

Written informed consent was obtained from the patient for participation and publication of this case report and any accompanying images. A copy of the written consent is available for review by the Editor‐in‐Chief.

## Conflicts of Interest

The authors declare no conflicts of interest.

## Data Availability

The data that support the findings of this study are available from the corresponding author upon reasonable request.
